# Perforin evolved from a gene duplication of MPEG1, followed by a complex pattern of gene gain and loss within *Euteleostomi*

**DOI:** 10.1186/1471-2148-12-59

**Published:** 2012-05-02

**Authors:** Michael E D’Angelo, Michelle A Dunstone, James C Whisstock, Joseph A Trapani, Phillip I Bird

**Affiliations:** 1 Department of Biochemistry and Molecular Biology, Monash University, Clayton, Melbourne, VIC, 3800, Australia; 2 The ARC Centre of Excellence in Structural and Functional Microbial Genomics, Monash University, Melbourne, VIC, 3800, Australia; 3 Department of Microbiology, Monash University, Melbourne, VIC, 3800, Australia; 4 Cancer Immunology Program, Peter MacCallum Cancer Centre, St Andrew’s Place, East Melbourne, VIC, 3002, Australia; 5 Department of Microbiology and Immunology, The University of Melbourne, Parkville, VIC, 3010, Australia

## Abstract

**Background:**

The pore-forming protein perforin is central to the granule-exocytosis pathway used by cytotoxic lymphocytes to kill abnormal cells. Although this mechanism of killing is conserved in bony vertebrates, cytotoxic cells are present in other chordates and invertebrates, and their cytotoxic mechanism has not been elucidated. In order to understand the evolution of this pathway, here we characterize the origins and evolution of perforin.

**Results:**

We identified orthologs and homologs of human perforin in all but one species analysed from *Euteleostomi,* and present evidence for an earlier ortholog in *Gnathostomata* but not in more primitive chordates. In placental mammals perforin is a single copy gene, but there are multiple perforin genes in all lineages predating marsupials, except birds. Our comparisons of these many-to-one homologs of human perforin show that they mainly arose from lineage-specific gene duplications in multiple taxa, suggesting acquisition of new roles or different modes of regulation. We also present evidence that perforin arose from duplication of the ancient MPEG1 gene, and that it shares a common ancestor with the functionally related complement proteins.

**Conclusions:**

The evolution of perforin in vertebrates involved a complex pattern of gene, as well as intron, gain and loss. The primordial perforin gene arose at least 500 million years ago, at around the time that the major histocompatibility complex-T cell receptor antigen recognition system was established. As it is absent from primitive chordates and invertebrates, cytotoxic cells from these lineages must possess a different effector molecule or cytotoxic mechanism.

## Background

Cytotoxic lymphocytes (CLs) is a collective term for natural killer (NK) and cytotoxic T lymphocytes (CTL). As the name suggests, these cells are cytotoxic towards virally infected, neoplastic or foreign cells. The two major mechanisms they use to elicit apoptosis in target cells involve (1) cell surface death receptors and their ligands (e.g., Fas/Fas-ligand) and (2) the granule-exocytosis pathway [1]. The latter involves the targeted secretion of specialised secretory lysosomes (granules) from CLs into the immunological synapse, a cleft formed at the site of CL-target cell contact [2]. The granules contain the granzyme family of serine proteases, effectors that cleave cytoplasmic proteins to induce apoptosis, and perforin, a membrane pore forming protein that is required for entry of the granzymes into target cells [3,4].

CTL form part of the adaptive immune system in all jawed vertebrates but not in earlier chordates [5,6]. The lamprey, a jawless vertebrate, has an unconventional adaptive immune system which does not use the major histocompatibility complex (MHC) or T cell receptor (TCR) recognition system, but a more primitive leucine rich repeat-containing antigen receptor [7]. This species appears to have CTL-like leukocytes but whether they are armed with granule mediated cytotoxic machinery is unknown. NK cells, by contrast, are more difficult to define than CTL, but appear to have evolved earlier. There is evidence for cells with NK properties in the tunicate *Botryllus schlosseri*, and the *Ciona intestinalis* genome includes homologs of some NK cell receptors [5,8]. More basic cytotoxic NK-like cells have been described in earlier divergent invertebrates such as earthworms [9]. How similar these cells are to conventional mammalian NKs, including their mechanisms of killing, remains to be seen.

Perforin (gene symbol *PRF1*) is essential and central to the granule-exocytosis pathway in mammals. Effective CL induction of apoptosis requires both granzymes and perforin, although at high concentrations perforin alone can kill cells by causing necrosis, whereas granzymes are ineffective without perforin to translocate them into the target cell cytoplasm. This is highlighted by the human autosomal recessive disease familial hemophagocytic lymphohistiocytosis type 2 (FHL2), caused by mutations in the perforin gene [10]. CTL from these patients cannot kill Fas-deficient target cells and so do not have an active granule-exocytosis pathway [10].

Perforin forms circular pores in the plasma membrane of target cells by a mechanism involving at least three steps: (1) perforin monomers bind to the membrane via their C2 domains in a calcium dependent manner; (2) monomers polymerise into a ring, mediated in part by salt bridging between residues in adjacent N-terminal membrane attack complex/perforin (MACPF) domains; (3) two clusters of α-helices within each MACPF domain rearrange into anti-parallel β-strands that puncture and span the membrane, creating an aqueous pore [11-16]. The mechanism of (3) and the order of (2) and (3) are inferred from structural similarity to the well-studied cholesterol-dependent cytolysin family of proteins as well as experimental observations of the perforin pore [16,17].

The MACPF domain has been identified in 12 human proteins and is named after the six best characterised members found in the immune system: five of the terminal complement components (C6, C7, C8α, C8β and C9) that form the membrane attack complex (MAC), and perforin [18,19]. The MAC is formed when C5b, C6, C7, C8 (a complex of C8α, C8β and C8γ) assemble on foreign cell membranes, which then recruits multiple C9 monomers to polymerise and insert into the membrane [20,21]. Perforin has long been compared to C9 as they are both able to polymerise and insert into membranes and the pores formed look similar by transmission electron microscopy [22-24].

The only other MACPF domain-containing protein known to be involved in the human immune system is macrophage expressed gene 1 protein (also referred to as mps1 and mpg-1, here-in the gene and protein is abbreviated as MPEG1), produced by macrophages [25]. Besides the MACPF domain MPEG1 contains one or more additional domains with no identified relationship to known protein folds, and a C-terminal transmembrane anchor [19]. MPEG1 is an ancient gene with homologs in species from one of the earliest metazoan lineages, the phylum *Porifera* (sponges), *Amphimedon queenslandica* and *Suberites domuncula*[26,27]. The homolog from *S. domuncula* is the best studied MPEG1 gene and is part of an ancient toll-like receptor pathway that is upregulated by lipopolysaccharide [26]. This role in innate immunity, along with its expression in macrophages, has led to the hypothesis that MPEG1 clears phagocytosed Gram-negative bacteria [28]. Indeed, recent evidence shows that the isolated MACPF domain from MPEG1 of the Pacific oyster *Crassostrea gigas* has anti-microbial activity against both Gram-positive and Gram-negative bacteria [29].

Here we trace the origins and evolution of the perforin gene to gain insight into the evolution of the granule-exocytosis pathway. Using a variety of approaches including linked gene comparisons, BLAST searches and protein phylogenetic trees we have catalogued all of the available perforin homologs. These data suggest that the perforin-dependent granule-exocytosis pathway originated in jawed vertebrates (*Gnathostomata*), at around the same time as true CTLs. In addition, we present evidence that MPEG1 is the precursor of perforin.

## Methods

### Identification of human perforin homologs

To search for perforin homologs, the human perforin protein sequence [Refseq:NP_005032.2] was used to query genome and protein databases on NCBI and ENSEMBL using both tBLASTn and BLASTp [30]. Hits representing true perforin homologs were distinguished from other MACPF domain-containing proteins/genes in two ways. Firstly, full-length sequences were subjected to domain prediction in PFAM, any sequences not possessing both a MACPF domain and C2 domain were discarded. Secondly, partial sequences without sufficient data to reasonably contain both domains were used to query the non-redundant protein database by BLAST, any sequences with top hits to proteins other than perforin were discarded. In some cases, annotated protein sequences in ENSEMBL required amendment to fix non-canonical splice sites and obtain valid start and/or stop codons. A complete table of accessions used in this study is in Additional file 1: Table S1, and a complete file of amended protein sequences used in this study is in Additional file 2: Figure S1.

### Locus diagrams

Genome scaffolds were viewed in ENSEMBL and NCBI and redrawn for ease of comparison. Orientation of genes were made relative to perforin, which was always represented in the positive orientation, except where it was necessary to be drawn in the negative orientation for comparison to other scaffolds in the figure.

### Assembly of *Ornithorhynchus anatinus* contigs

We exported the contig sequences containing perforin genes and used these as queries with megaBLAST on the trace archive (*Ornithorhynchus anatinus* – other) to search for end sequences of BAC and fosmid clones. We used the paired ends of matching clones to query the reference genome sequence to find matching contigs and by comparing the orientation of the clone hits we could infer the orientation of the various contigs. We assembled ten previously disconnected contigs in one instance, and two contigs in another instance, shown in Additional file 3: Figure S2. To search for genes on these contigs we viewed them in ENSEMBL. We found annotated partial genes that matched ADAMTS14 on some contigs, and where there were no annotated genes we searched for homologous protein sequences using tBLASTx and compiled these into a single protein sequence in the order suggested by our assembly.

### Protein sequence alignment and phylogenetic trees

Fish perforin protein sequences were aligned using ClustalW and manually edited to minimise gaps and align conserved structural elements in bioedit (version 7.0.5.3) [31,32]. The alignment is available in Additional file 4: Figure S3. The Bayesian inference tree was constructed using MrBayes (version 3.2.1) with the WAG amino acid substitution model, invariant sites and a gamma distribution [33,34]. 100,000 generations were run with trees sampled every 100 generations and the final 50% majority rule tree was calculated after discarding the first 25% of trees as burnin. Trees were displayed using FigTree (version 1.3.1) [35].

To align the MACPF domains of perforin, C6 and MPEG1, domain boundaries were chosen based on three rounds of PSI-BLAST using full-length murine MPEG1 as the probe, together with information of the perforin and C8 structures [16,36]. Sequences truncated to the MACPF boundaries were then aligned with ClustalW, positions with gaps were removed and phylogenetic trees were constructed with MrBayes as above. The alignment (including gaps) is available in Additional file 5: Figure S4. Phylogenetic trees have been deposited in TreeBASE and can be accessed here: http://purl.org/phylo/treebase/phylows/study/TB2:S12411.

## Results

### Search criteria for perforin orthologs

**Figure 1:**
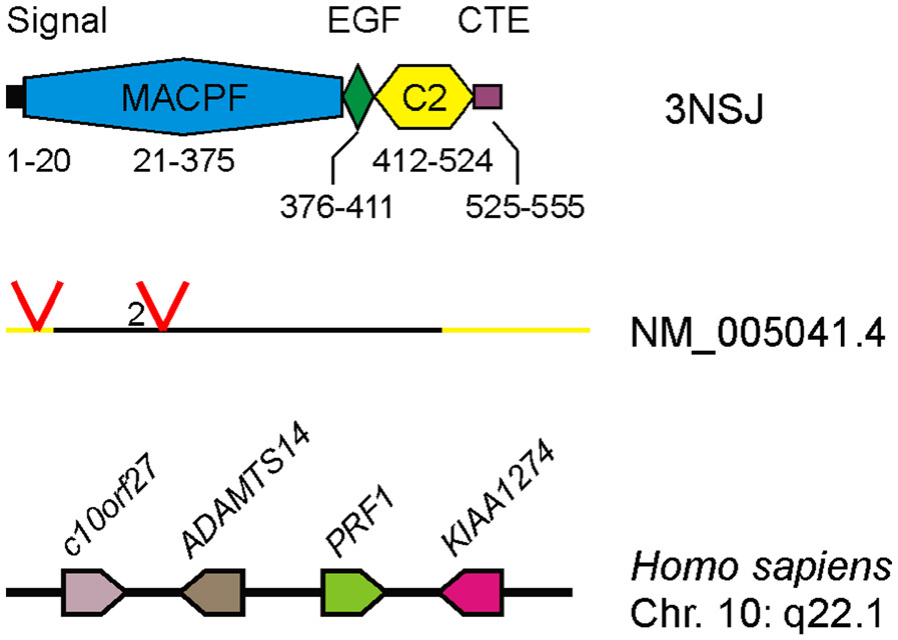
**Human perforin protein domains, transcript structure and genetic locus.** The protein domains are shown along with the amino acids numbers they encompass as inferred from the mouse perforin structure [PBD:3NSJ]. Signal, secretion signal peptide; MACPF, membrane attack complex/perforin domain; EGF, epidermal growth factor-like domain; C2, C2 domain; CTE, c-terminal extension. The transcript [Refseq:NM_005041.4] is represented as a line where 1 cm = 500 bp, the black region represents the coding sequence (CDS), the yellow region represents the untranslated regions and the red ‘V’ shapes indicated positions where introns have been spliced out. The phasing of the CDS intron is indicated to left of the marker. The genes on *Homo sapiens* chromosome (Chr) 10 q22.1 (drawn as a black line, not to scale) are shown as arrowheads, with their gene symbols above. The direction of the arrowhead indicates the relative transcriptional orientation.

To search for orthologs of human perforin we first examined and set defining criteria based on the human perforin protein, mRNA transcript and gene locus (Figure 1). Perforin contains a MACPF, EGF-like and C2 domain, and with the X-ray crystal structure of mouse perforin recently being solved, the domain boundaries have been clearly delineated [16]. The combination of a MACPF and EGF-like domain is seen in many MACPF proteins, however, to date, the C2 domain is unique to perforin and thus was a key criterion for our ortholog searches. The perforin gene structure is also unique among human MACPF family genes, none of which share perforin’s 3 exon, 2 intron splicing pattern. The first intron, located in the 5’ untranslated region (UTR) can only be tracked where expressed sequence tag (EST) information is available. The other intron, found in the coding sequence (CDS), is conserved with identical phasing (phase 2) in mammalian perforin orthologs. Human perforin is a single copy gene located at q22.1 on chromosome 10 and is flanked by the genes c10orf27, ADAMTS14 and KIAA1274 (Figure 1).

### Conservation of the human perforin locus extends to the last common ancestor (LCA) of *Mammalia* but not to earlier vertebrate lineages

**Figure 2:**
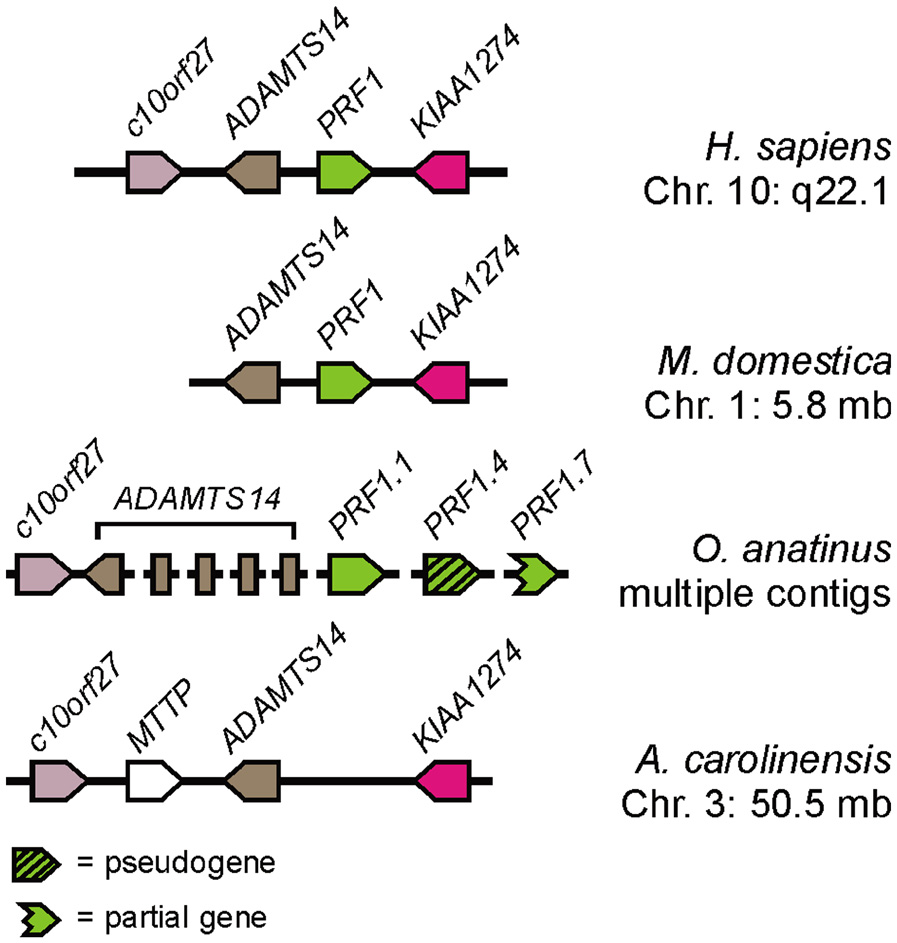
**Conservation of the human perforin locus extends to platypus but not more divergent vertebrates.** Genome scaffolds/contigs are drawn as a black lines (not to scale), and genes are shown as arrowheads, with their gene symbols above. Syntenic genes are color coded. The direction of the arrowhead indicates the relative transcriptional orientation, and the relevant chromosome (Chr) coordinates are indicated on the right.

**Figure 3:**
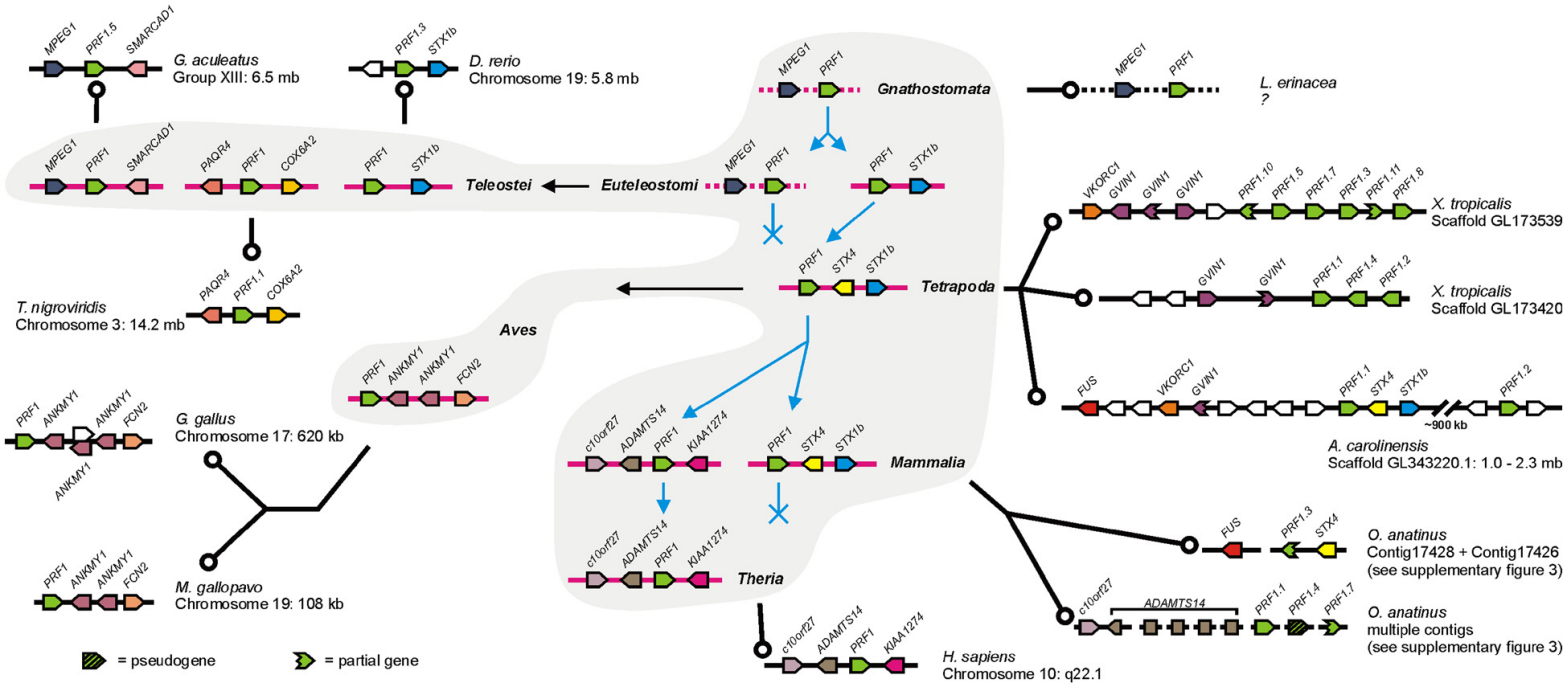
**A roadmap of perforin gene evolution created by tracking the perforin gene locus through extant vertebrate genomes.** Scaffolds from the last common ancestors of vertebrate lineages are shown as pink lines (not to scale), with a grey background. Genome scaffolds from representative extant species are drawn as a black lines (not to scale), with a white background. Predicted scaffolds (where direct evidence is not available) are shown as dotted lines. Genes are shown as arrowheads, with their gene symbols above. Syntenic genes are color coded; genes that are not conserved are white with no label. The direction of the arrowhead indicates the relative transcriptional orientation and the relevant genome coordinates indicated on the right.

We followed the synteny of this locus and found the position and orientation of these three genes was conserved throughout *Theria* (placental mammals and monotremes) (Figure 2). Looking at more divergent vertebrates such at the reptile *Anolis carolinensis* we observed that the position and orientation of the other genes of this locus were conserved but perforin was no longer present. A similar situation was evident in the bird *Gallus gallus* and fish *Takifugu rubripes*. The data available from assembled genomes thus points to the appearance of perforin at this locus some time between the last common ancestors (LCA) of *Amniota* and *Theria*. To investigate this we looked at the monotreme *O. anatinus* (platypus). Using the human perforin protein sequence we used tBLASTn to query the platypus genome on ENSEMBL and found multiple full length and partial matches including some that appeared to be pseudogenes. These were mostly on short contigs with no other genes present. Given the importance of this question we assembled this region ourselves (see methods). Using this approach we assembled 10 contigs (Additional file 3: Figure S2) and could now search these for genes predicted to be linked to perforin. We identified protein fragments with similarity to ADAMTS14 on all of the linked contigs (see methods). Combining the protein fragments from these contigs in the order suggested by our assembly (Additional file 3: Figure S2) and using BLASTp we found ADAMTS14, affirming the order of our *de novo* assembly. C10orf27 was also found on one of these linked contigs, further strengthening the match between the platypus and human loci (Figure 2, Additional file 3: Figure S2). This indicates a new phylogenetic position for the appearance of the perforin gene at this locus, between the LCA of *Amniota* and *Mammalia* (Figure 3).

### Perforin is present in *Gnathostomata* but not earlier *Chordata*, and many species have multiple perforin genes

**Figure 4:**
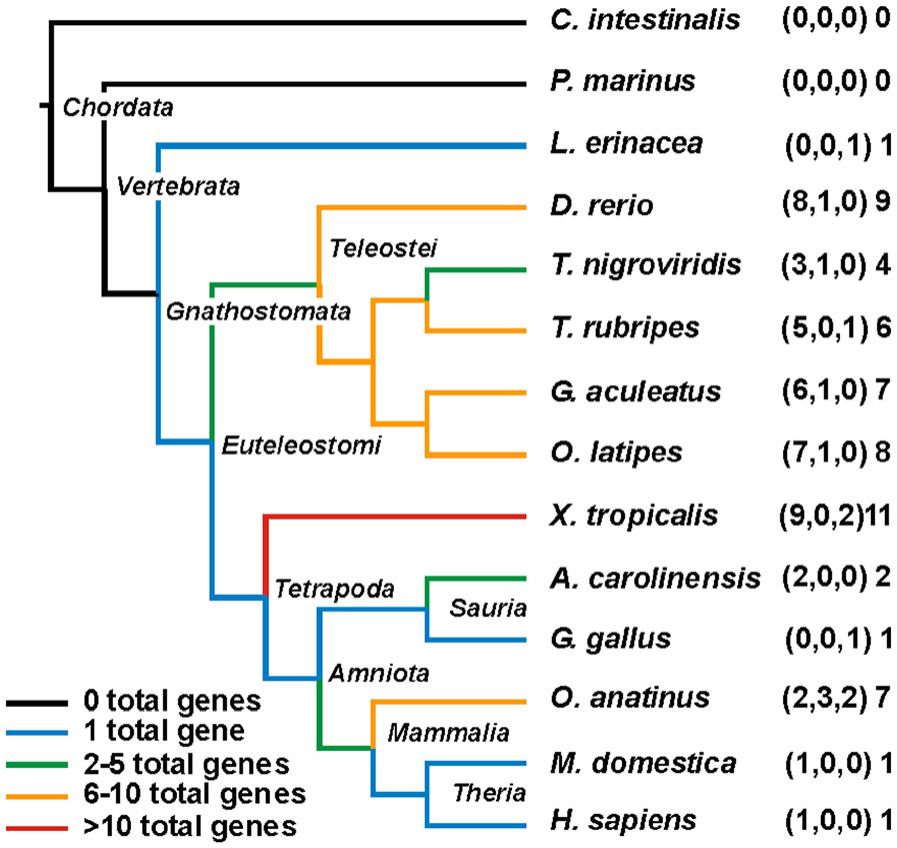
**Perforin gene distribution in*Chordata*.** The phylogenetic tree shows the relationship between the species. The numbers of genes per species is shown on the right, in the format: (# full length genes, # partial genes, # pseudogenes) # total genes. Line colour reflects total gene number as per the key.

As perforin is absent from this locus in earlier vertebrate lineages we subsequently changed our search strategy to using tBLASTn with the human perforin protein sequence to search the nucleotide databases in NCBI and ENSEMBL and BLASTp to search the protein databases in NCBI. Using this approach we found perforin genes in all species of bony vertebrate (*Euteleostomi*) with available data, with the exception of zebra finch (see discussion). The only evidence of a perforin gene in earlier *Gnathostomata* was a single EST from *Leucoraja erinacea* (little skate) which matched part of the human perforin protein by BLASTx. The only species from the class *Chondrichthyes* with available genome resources, the elephant shark (*Callorhinchus milii*), does not show any evidence of a perforin gene but due to the low sequence coverage (1.4×) we cannot conclude that it is absent. Earlier chordates such as the tunicate *C. intestinalis* and lamprey (*Petromyzon marinus*) show no evidence of a perforin ortholog, nor do any earlier *Metazoa* (Figure 4). This suggests that the perforin gene originated about 500 million years ago before the divergence of *Chondrichthyes* and *Euteleostomi* (Figure 3) [37].

Tracing perforin linked genes through bony vertebrate lineages we created maps of perforin loci and inferred the locus configurations in various major lineages (Figure 3). This analysis shows that the perforin gene has undergone a striking degree of duplication and repositioning within vertebrate genomes, being found at no fewer than 5 distinct loci across various lineages, and more if individual fish species are considered. The most conserved locus is the STX1b locus, which emerged before the LCA of *Euteleostomi*, as it is found in both teleost fish and tetrapods (Figure 3). This locus is present in amphibians and reptiles but absent in birds and therian mammals. It is also present in platypus, where we again linked two contigs using the approach described above (Figure 3, Additional file 3: Figure S2). The orientation of the *O. anatinus* perforin 1.3 gene relative to STX4 is opposite to other examples of this locus but this may be a result of incorrect assembly rather than a true gene inversion as there are many gaps in this short contig. The second contig contains the FUS gene, present at this locus in *A. carolinensis* which further confirms the arrangement of this locus in the platypus.

In Figure 4 we have summarised the numbers of full length genes, apparent pseudogenes and partial genes present in the available bony vertebrate genomes. All therian mammals have a single perforin gene and no pseudogenes, with the exception of the hedgehog (*Erinaceus europaeus*), which also has a full length pseudogene, and the hyrax (*Procavia capensis*) which also has a partial pseudogene. By contrast, most other lineages within *Euteleostomi* have species with multiple perforin genes. Birds appear to be an exception with evidence for only one perforin gene in each of chicken (partial), turkey (possible pseudogene, see discussion) and mallard duck (partial) genomes, and the aforementioned exception of zebra finch (no perforin gene). All sequenced teleost fish have at least three full length perforin genes and the frog *Xenopus tropicalis* has the largest number of perforin genes at 11. We investigated these cases of perforin gene duplication in more detail.

### The LCA of *Teleostei* possessed multiple perforin genes

We compiled all the full length perforin protein sequences from the five available fish genome sequences (*Danio rerio, Takifugu rubripes, Tetraodon nigroviridis, Gasterosteus aculeatus and Oryzias latipes*) as well as nine sequences from cloning projects from five other fish species (*Paralichthys olivaceus, Oncorhynchus mykissSalmo salar, Ctenopharyngodon idella* and *Carassius auratus langsdorfii*) giving a total of 38 full length fish perforin proteins [38-41].

**Figure 5:**
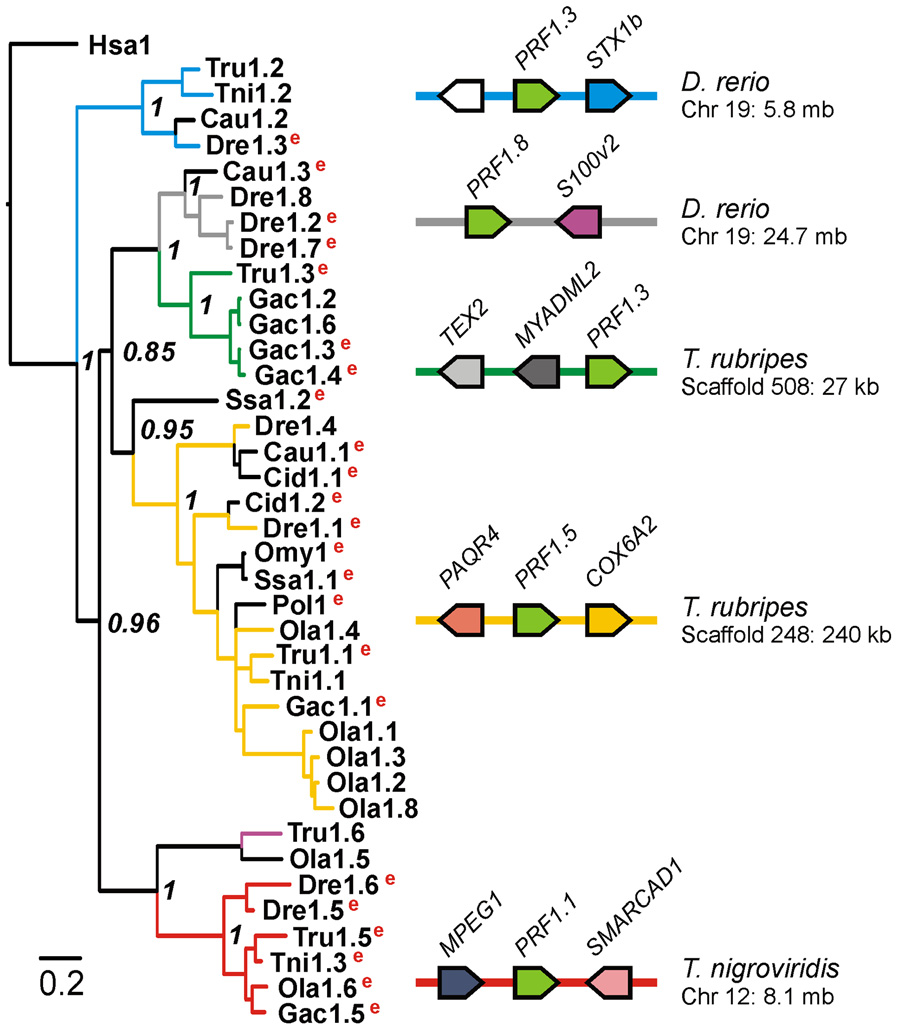
**Multiple perforin genes were present at distinct loci in the LCA of teleost fish.** The Bayesian inference phylogenetic tree of fish perforin homologs was made using the alignment in Additional file 4: Figure S3. Posterior probabilities for major clades are shown in italics. Sequences with evidence of expression are denoted by the letter ‘e’ to the right of relevant tip labels. The clades formed reflected the loci to which genes belong, as shown by a representative scaffold to the right of each clade. Related branches and scaffolds are color coded. Proteins are labelled as the three letter genus/species abbreviation followed by the relevant perforin gene number. Genus/species abbreviation are: *Homo* sapiens, Hsa;* Takifugu rubripes*, Tru; *Tetraodon nigroviridis*, Tni; *Danio rerio*, Dre; *Carassius auratus langsdorfii*, Cau; *Oryzias latipes*, Ola; *Gasterosteus aculeatus*, Gac; *Salmo salar*, Ssa; *Ctenopharyngodon idella*, Cid; *Oncorhynchus mykiss*, Omy; *Paralichthys olivaceus*, Pol.

To examine the relationship between these proteins we constructed a phylogenetic tree (Figure 5). We expected that the clades formed would provide insight into the evolution of fish perforin paralogs. Clades containing proteins from the same species would arise from species-specific duplications, and comprise recently derived paralogs. Conversely, clades containing proteins from different species comprise orthologs arising during speciation. In the tree in Figure 5 we see both types of clades formed, for example the sub-clade containing *O. latipes* perforin 1.1, 1.2, 1.3 and 1.7 is made up of recently derived paralogs, and the clade containing *D. rerio* perforin 1.3, *T. nigroviridis* perforin 1.2, *T. rubripes* perforin 1.2 and *C. auratus langsdorfii* perforin 1.2, which have arisen by speciation.

We also noted that each clade grouped genes found at syntenic loci. Two clades, with genes at PAQR4 and MPEG1 loci (termed here ‘PAQR4 clade’, ‘MPEG1 clade’ etc.), have representatives in all fish genome data available, demonstrating that they were present in the LCA of *Teleostei* (Figure 3). Others have representatives in only a few species, suggesting that they either arose via a later duplication or that they were present in the LCA of *Teleostei* but were subsequently lost in some lineages. We see evidence for both of these processes. For example (1) the MYADML2 clade is present in *T. rubripes* and *G. aculeatus* but no other species, suggesting it was acquired in the *Percomorpha* lineage and subsequently lost from *T. nigroviridis* and *O. latipes*; and (2) the STX1b clade is found in *D. rerio*, *T. rubripes* and *T. nigroviridis* as well as some tetrapods (showing that it must have been present in the LCA of *Teleostei*) but is lost from *Smegmamorpha*.

There are also nine perforin sequences from fish species without genome assemblies on this tree (black branches, Figure 5). This allowed us to make predictions about the loci of these genes based on their clustering. Of these, six fall in the PAQR4 clade and one in the STX1b clade. *C. auratus langsdorfii* perforin 1.3 clusters with a group of linked *D. rerio* perforins (1.2, 1.7 & 1.8) at the S100v2 locus, a locus which we originally believed to be *D. rerio* specific but this additional evidence suggests that it may have originated earlier, possibly in the LCA of the *Cyprinidae* family. The other sequence, *S. salar* perforin 1.2 does not make a strong cluster with any group and may represent a new locus, perhaps specific to the Atlantic salmon.

We were interested to know about the expression of these multiple fish perforin genes. We cross-referenced our collection of perforin genes with available EST data. In this way we found evidence that seventeen fish perforin genes are expressed in a wide range of tissues, and that more than one perforin gene is expressed in most species (Figure 5). Looking at how these expressed genes clustered we noticed that while all major clades contained genes that were expressed in one or more species, only for members of the MPEG1 clade was expression evident in all species examined. It is tempting to speculate that this locus contains a highly - or widely - expressed perforin gene (which would lead to a high probability of representation in EST datasets). Conflicting with this is the fact that most of the manually cloned perforin genes fall within the PAQR4 clade and none fall in the MPEG1 clade. In any case these findings raise the interesting question of why fish possess multiple perforin paralogs.

**Figure 6:**
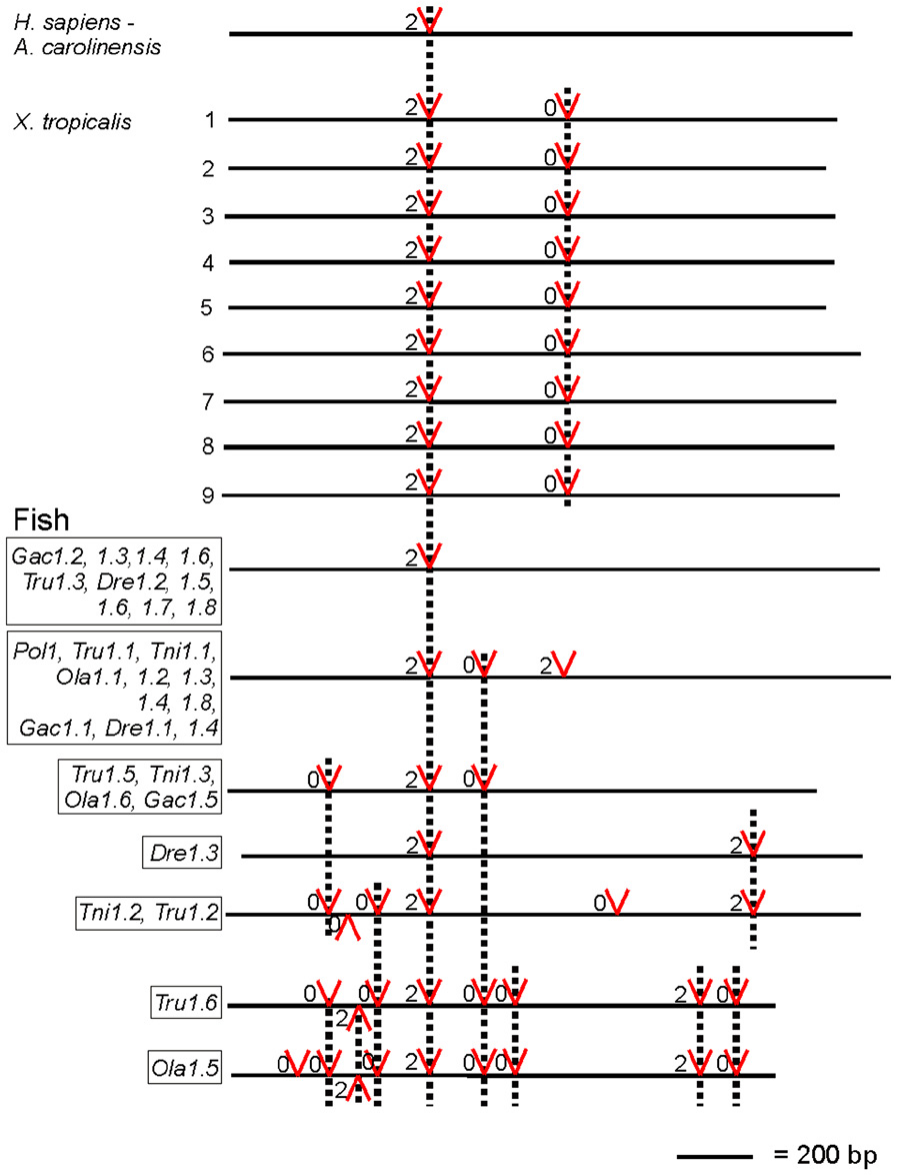
**Perforin genes have acquired introns in multiple lineages.** The coding sequences (CDS) of perforin genes are drawn as black lines to the scale shown. Positions of introns are indicated by red ‘V’ shapes, with the intron phasing numbered at the left of the markers. Conserved introns are linked with dotted vertical lines. Introns in the 5’ untranslated region (UTR) are not shown as they require EST evidence and therefore cannot be traced consistently.

To further classify fish perforin genes we analysed their exon/intron patterns and phasing. Where 5’ ESTs were available we noted an intron in the 5’UTR, as seen in mammalian perforin genes (data not shown). In stark contrast to mammalian and reptile perforin genes, many of the fish genes have gained between one and eight introns (Figure 6). These patterns are related but cannot be used to infer the descent of these paralogs as they indicate that both intron gain and loss has occurred. For example, the first intron of *Tru1.5* is conserved in *Tni1.2* but not *Tru1.1*, while the third intron of *Tru1.5* is conserved in *Tru1.1* but not *Tni1.2*, so we cannot deduce which intron was gained first. Also, one of these introns must have been lost in the duplicated genes but we cannot determine which one. While there are many different exon/intron patterns, some genes still have the same, simple pattern as the mammalian and reptile perforin gene. This implies that the LCA of *Euteleostomi* had a perforin gene with this structure (a single phase 2 CDS intron), and that some fish genes have subsequently acquired additional introns.

### The additional perforin genes in *X. tropicalis* are recent, lineage specific duplications

The massive expansion of perforin genes in *X. tropicalis* intrigued us. As shown in Figure 3, nine of these are found on two scaffolds. Both of these are likely to be STX1b loci, supported by upstream gene information in comparison to *A. carolinensis*, and both scaffolds finish shortly after perforin genes and are missing downstream sequence that would confirm this hypothesis. The remaining two are all found alone on small scaffolds with no linked genes to provide context.

To further assess the origin of these multiple genes we looked at their exon/intron patterns. All of the nine full length genes in *X. tropicalis* contain a second CDS intron (phase 0) in addition to the completely conserved phase 2 intron (Figure 6). This intron is specific to this species and demonstrates conclusively that these multiple perforin genes are a result of lineage specific duplications, occurring subsequent to intron gain. Genome information from additional amphibians would allow us to narrow the timeframe of this event but at this stage we can only conclude that it occurred sometime after the divergence of *Amphibia* and *Amniota*. EST evidence exists for two full length and one partial gene, again raising the question of why some species need many perforin genes when one is sufficient in mammals.

### Perforin most likely evolved from an MPEG1-like ancestor

**Figure 7:**
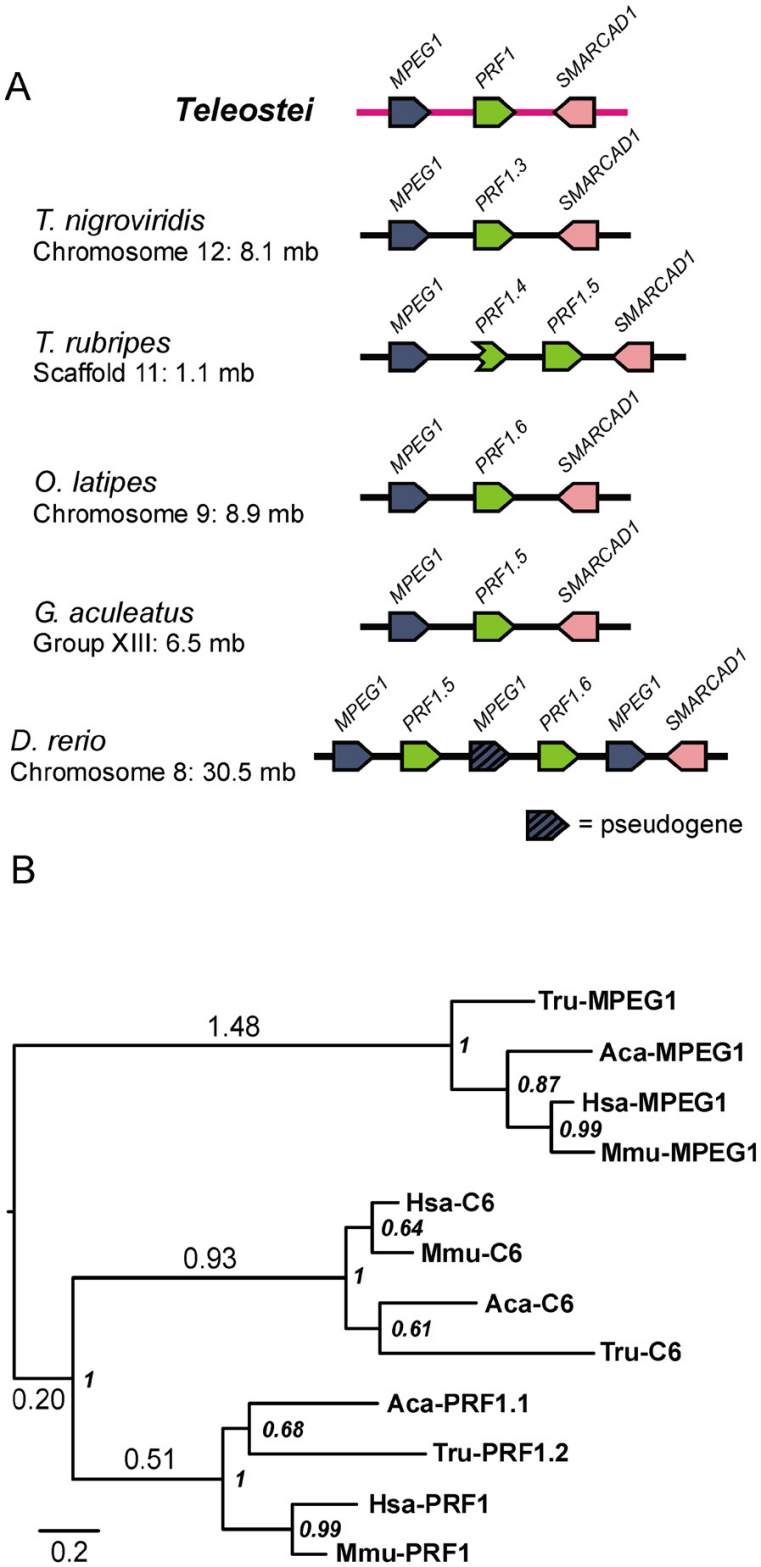
**Genomic evidence shows that perforin originated from a duplication of MPEG1, but the MACPF domain of perforin is more similar to C6. A**. Genome scaffolds from the five fish species with assembled genomes that contain both MPEG1 and perforin genes are shown, along with the inferred scaffold of the last common ancestor (LCA) of *Teleostei*. **B**. Bayesian inference phylogenetic tree generated from an alignment of the MACPF domains of perforin, MPEG1 and C6 from human (Hsa), mouse (Mmu), anole lizard (Aca) and fugu (Tru). The tree is rooted at the midpoint. Node labels are posterior probabilities (italicised). Important branch lengths are labelled to indicate the degree of divergence between the three proteins.

While performing our locus analysis of fish perforin genes we discovered the gene MPEG1 is adjacent to perforin and transcribed in the same orientation at one locus in each fish genome (Figure 3 and Figure 7a). This is the only MACPF family gene found in close proximity to perforin, and is apparent in the earliest lineage that has perforin genes (with the exception of *L. erinacea*, which currently lacks genome resources). This indicates that perforin could have originated from a segmental duplication by uneven crossing-over in the region of the MPEG1 gene. We therefore looked at the exon/intron patterns for additional evidence. The single phase 2 CDS intron in perforin is 100% conserved in perforin genes, and occurs in the region encoding the MACPF domain. We anticipated that this would be conserved in another MACPF gene, but no other genes we examined contain this intron; it is a unique and defining characteristic of perforin genes. Instead we looked at the numbers of CDS introns in vertebrate complement, perforin and MPEG1 genes. We summarised the CDS intron numbers of these genes from *D. rerio* from ENSEMBL and saw that MPEG1 and perforin have just one while all of the complement genes have at least 10 (Table 1). This prompted us to examine the MPEG1 intron in more detail. All fish MPEG1 genes have this conserved intron, but tetrapod MPEG1 genes have no CDS introns, and neither do earlier, invertebrate MPEG1 genes (from *Lottia gigantea* and *A. queenslandica*). We concluded that the MPEG1 gene has gained an intron in the *Teleostei* lineage, but it exists as a continuous open reading frame in other lineages. This simpler configuration makes MPEG1 more likely than the complement genes to be the precursor of perforin. An alternate, more complicated explanation is that one of the complement components have lost multiple introns and/or given rise to perforin from a retrotransposed processed transcript.

**Table 1:** Zebrafish complement, perforin and MPEG1 intron numbers

**Gene Symbol:**	**CDS introns:**	**Accession:**
PRF1.6	1	ENSDARG00000024522
MPEG1^a^	1	ENSDARG00000057113
C6^a^	16	ENSDARG00000057121
C7^a^	17	ENSDARG00000039516
C8a	10	ENSDARG00000039517
C8b	11	ENSDARG00000016319
C9	10	ENSDARG00000055290

^a^These genes have paralogs with an identical/similar number of introns.

To further examine the evolutionary relationship between perforin, MPEG1 and complement proteins we curated and aligned these protein sequences from human, mouse, lizard and fugu, and produced a phylogenetic tree. The terminal complement components and perforin all contain an EGF-like domain after their MACPF domain. While MPEG1 has a cysteine rich region in a similar position, this is not believed to be an EGF-like domain [25]. We therefore restricted our alignment to the MACPF domain, the boundaries of which were chosen based on PSI-BLAST results and structural data and then aligned with ClustalW. As shown in Figure 7b, the branch lengths of the tree show that the MACPF domain of perforin is more closely related to C6 than to MPEG1. This is also true of trees where C6 is substituted for C7 (data not shown).

**Figure 8:**
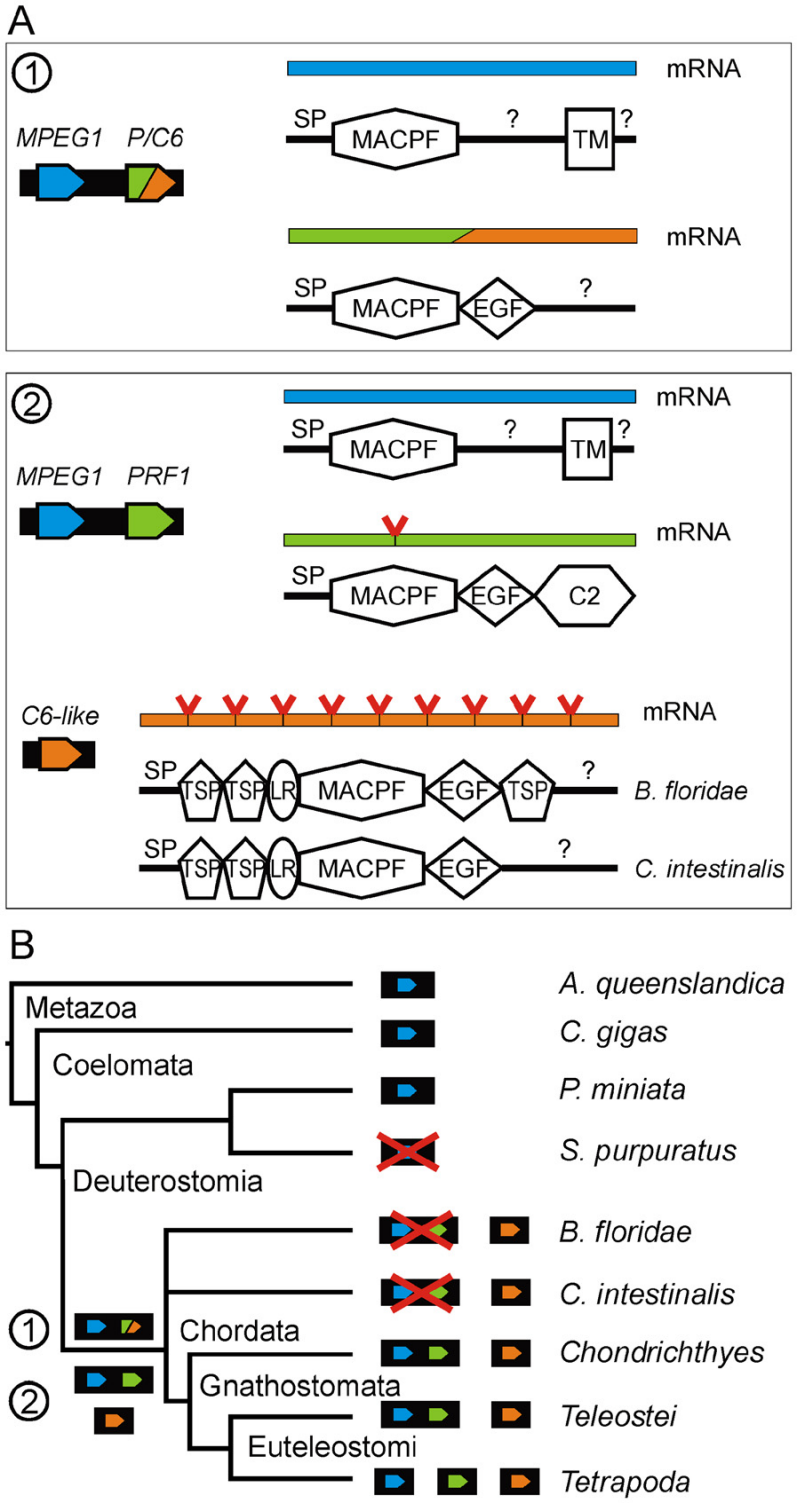
**Predicted events in the evolution of perforin: A**. Locus, transcript and protein domain architectures of: i. the gene cluster of MPEG1 and a hypothetical perforin/C6 common ancestor (P/C6); ii. the MPEG1, perforin gene cluster and a C6-like gene from early chordates. Domains are abbreviated as follows: secretion signal peptide, SP; membrane attack complex/perforin, MACPF; transmembrane anchor, TM; epidermal growth factor-like, EGF; C2 domain, C2; thrombospondin, TSP; low-density lipoprotein-receptor class A, LR; unknown region, ?. **B**. Phylogenetic tree showing the relationship of species from major branches of *Metazoa*. The loci for MPEG1, perforin and C6 are shown and loci not found in some genomes are crossed out. Genus/species abbreviations are:* Amphimedon queenslandica, A. queenslandica; Crassostrea gigas*, *C. gigas*; *Patiria miniata*, *P. miniata*; *Strongylocentrotus purpuratus*, *S. purpuratus*; *Branchiostoma floridae*, *B. floridae*; *Ciona intestinalis*, *C. intestinalis*.

Considered together, the genomic and protein evidence suggests that an ancient MPEG1 gene underwent a local duplication to produce a common ancestor of perforin and C6 (Figure 8). This precursor retained the MPEG1 gene structure but eventually lost the transmembrane anchor and gained an EGF-like domain. The precursor then duplicated to a distant locus. Subsequently, the paralog linked to MPEG1 evolved into perforin by gaining the conserved intron and a C2 domain, explaining the linked MPEG1 and perforin genes evident in extant fish species. The other paralog gave rise to the C6-like genes, as seen in the early chordates *C. intestinalis* and *Branchiostoma floridae*, hypothesised to be the common ancestors of C6-9 [5,42,43]. This involved acquiring multiple TSP domains and an LDLRA domain, as well as at least 9 introns (Figure 8a). The evolution of a C6-like gene must have been completed by the LCA of *Chordata* as species from multiple lineages possess this gene (Figure 8b). The MPEG1/perforin locus has either been lost from the species *C. intestinalisB. floridae* and *P. marinus*, or is not covered by these genome projects (Figure 8b). By the LCA of *Gnathostomata*, MPEG1, perforin and C6-9 were all established and by the LCA of *Tetrapoda* MPEG1 and perforin were no longer linked genes (Figure 3, Figure 8b).

## Discussion

Perforin is a critical protein in the granule-exocytosis pathway of CLs, therefore tracing the evolution of this gene yields insights into the evolution of the pathway itself (discussed below). We have characterised all of the perforin genes from available databases and find many species with multiple perforin genes. Classifying these duplicated genes led to the discovery of multiple gene duplication events in different lineages, and insights into the origins of perforin itself.

### MPEG1: the precursor of perforin?

As no other proteins share the entire domain structure of perforin, the origin of the perforin gene is obscure. In terms of structure and function it has always been compared to C9 because this terminal complement component also polymerises and inserts into membranes, although unlike perforin it requires a primer (the C5b-C8 oligomer) to initiate membrane binding. Although the complement proteins appear to have descended from a common ancestral gene, a precursor of the perforin gene has not yet been identified [42].

We suggest here that perforin originated from the ancient gene MPEG1 on the basis of similar gene structure and chromosomal co-location of these genes in ancient bony vertebrate lineages. It is more likely that perforin originated from MPEG1 than from any other extant MACPF family member because no other genes encoding MACPF domain-containing proteins share these genetic characteristics. However, given that the MACPF domains of perforin and complement component C6 are more closely related than those of perforin and MPEG1, it is likely that perforin and MPEG1 are separated by an intermediate with features of both perforin and a terminal complement component. At present no available genome data contains remnants of such a gene.

The origins of MPEG1 itself are presently obscure, but its presence as an intronless gene in one of the most ancient metazoan lineages (e.g. *Porifera*), and absence in other eukaryotic kingdoms, coupled with the existence of MACPF proteins in prokaryotes, suggests a mechanism of horizontal gene transfer from prokaryotes to early metazoans.

### Do birds possess functional perforin genes?

Perforin genes appear in all available bony vertebrate genomes with the surprising exception of zebra finch (*Taeniopygia guttata*). The presence of other genes of the granule-exocytosis pathway in zebra finch (granzyme A), as well as perforin’s absolute conservation in *Euteleostomi*, predicts that any tetrapod without a perforin gene would be severely immunocompromised, yet there are no reports of zebra finches having such a defect. We therefore suggest that this species does indeed have a perforin gene but it has not yet been uncovered by sequencing projects. The turkey (*Meleagris gallopavo*) perforin gene also appears to be affected by a sequencing error; the gene contains a frameshift caused by a single nucleotide deletion. ENSEMBL corrects this by modelling a frameshift intron (2 bp) to maintain the coding sequence, but the possibility remains that this perforin gene in turkey is a pseudogene, in which case the species would be immunocompromised unless a functional paralog has been missed. The best studied bird species, the chicken (*G. gallus*) has only a partial perforin gene sequence annotated, with a gap in the assembly where the remainder of the gene should be found. Chicken perforin expression has been assessed in the context of Marek’s disease, where mRNA was shown by real time PCR to be upregulated in the spleens of infected chickens, although another study involving infectious bursal disease saw no upregulation in bursal mononuclear cells [44,45]. This suggests that at least one bird species has a functional perforin gene but ultimately more data, particularly EST or biological data, is required to make firm conclusions about the perforin gene in birds.

### Multiple perforin genes for multiple functions?

It is well established that a whole-genome duplication occurred in the *Teleostei* lineage, and that many genomic rearrangements are possible when reverting from a tetraploid to a diploid state [46,47]. Duplications of genes functioning in the immune system have been noted before in teleost fish, for instance an expansion of the CC chemokine genes has been described [48]. However perforin is an extreme example with a single gene in the *Gnathostomata* LCA giving rise to nine in *Danio rerio*. Paralogs are likely to have arisen from the whole-genome duplication and from local and distal segmental duplications. Multiple gene loss as well as intron gain and loss events have also occurred, highlighting the instability of the perforin gene in the teleost fish.

One of the most interesting questions that the duplicated fish perforin genes poses is why fish require so many, when in therian mammals a single gene is sufficient for the function of cytotoxic lymphocytes. Simple explanations of this phenomenon are that perforin has evolved additional molecular functions in fish (unlikely as the protein domains are highly conserved), and/or it has a broader tissue distribution and role or a more complex regulatory pattern than in mammals. For example, perforin is essentially restricted to two hematopoetic cell types in mammals (CTL and NK cells) and is controlled by similar circuitry. In fish it may be present in three or more immune cell types, with each paralog being restricted to a particular type. Alternatively, all perforin paralogs may be present in the same cell type, with each responding to distinct developmental or environmental signals. Indeed one study characterizing perforin from *C. auratus langsdorfii* notes that only one of the three perforin genes cloned was up-regulated by allo-reactive stimulation as measured by real time PCR, suggesting that the other two are not important for CTL cytotoxicity [41]. However it has been shown in mice that the perforin protein can be translated on demand from a stored pool of mRNA so increased mRNA expression may not be required to increase levels of perforin protein [49]. Nevertheless, the study by Nakanishi and colleagues, as well a wealth of EST data which we have mapped to our collection of perforin genes, demonstrates that multiple perforin genes per species are expressed at least at the mRNA level. Being present at distant loci, it is likely that these paralogs would be controlled by different regulatory elements, and therefore may have different tissue distribution. The role of these extra perforins will remain obscure until molecular and cellular studies examine their functions.

### Emergence of the perforin-mediated cytotoxic pathway

Cell-mediated cytotoxicity has been described in many species but the mechanisms of killing are not well understood except in mammals. Invertebrates can determine self-non-self and NK-like cells have been implicated in rejecting non-self cells [50]. In addition, a primitive form of adaptive immunity is present in the lamprey, a jawless vertebrate. This species possesses T cell-like lymphocytes that would be expected to kill target cells [51]. Our data suggests that ancient cytotoxic cells from species earlier than *Gnathostomata* are unlikely to have an active granule-exocytosis/perforin pathway, so invertebrate NK-like cells and lamprey CTL therefore must kill their targets in another fashion. This pathway seems to have arisen concurrently with the MHC-TCR antigen presentation system, suggesting primordial CTLs possessed perforin, whereas NKs acquired this cytotoxic machinery after their inception.

## Conclusions

The pore-forming protein perforin is the only component that is absolutely required for the granule-exocytosis pathway that cytotoxic lymphocytes deploy to eliminate deleterious cells. The perforin gene is present in *Gnathostomata* but not earlier species. Perforin evolved from a duplication of the related gene MPEG1, and shares a common ancestor with the terminal complement components. Surprisingly, we find that most bony vertebrate species predating placental mammals have multiple perforin genes, of unknown function. These findings also indicate that cytotoxic cells from invertebrates and jawless vertebrates must use alternative proteins or pathways to kill their target cells.

## Abbreviations

MHC = Major histocompatibility complex; TCR = T-cell receptor; MPEG1 = Macrophage expressed gene 1; CL = Cytotoxic lymphocyte; NK = Natural kill cell; CTL = Cytotoxic T lymphocyte; FHL2 = Familial hemophagocytic lymphohistiocytosis type 2; MAC = Membrane attack complex; MACPF = Membrane attack complex/perforin; C6 = Complement component 6; EGF-like = Epidermal growth factor-like; UTR = Untranslated region; EST = Expressed sequence tag; CDS = Coding sequence; LCA = Last common ancestor.

## Competing interests

The authors declare that they have no competing interest.

## Authors’ contributions

MED identified homologs, performed locus analyses, constructed sequence alignments and phylogenetic trees, helped design the study and drafted the manuscript. MAD constructed and interpreted sequence alignments and phylogenetic trees and helped conceive the evolutionary model. JCW helped conceive the evolutionary model and helped draft the manuscript. JAT helped draft the manuscript. PIB conceived and designed the study, interpreted results and drafted the manuscript. All authors read and approved the final manuscript.

## Supplementary Material

Additional file 1 Table S1. List of accessions used in this study. Click here for file

Additional file 2 Figure S1. Manually annotated perforin protein sequences used in this study. Click here for file

Additional file 3 **Figure S2. Platypus contig assembly by trace archive BLAST.** Contigs are shown in blue with arrows indicating their relative 5’–3’ orientation. Linking BACs and fosmid clones are shown in grey and white respectively, with red regions showing where clone end sequences match contigs. Genes are shown as arrowheads with gene symbols above. Click here for file

Additional file 4 **Figure S3. Alignment of fish perforin proteins for figure**5**tree.** Click here for file

Additional file 5 **Figure S4. Perforin, MPEG1 and C6 alignment for figure**7**tree.** Click here for file
