# Supplementary figures and images for: Perforin evolved from a gene duplication of MPEG1, followed by a complex pattern of gene gain and loss within Euteleostomi
Source: BMC Evol Biol. 2012 May 2;12:59. doi: 10.1186/1471-2148-12-59 (PMC3477005; doi:10.1186/1471-2148-12-59)

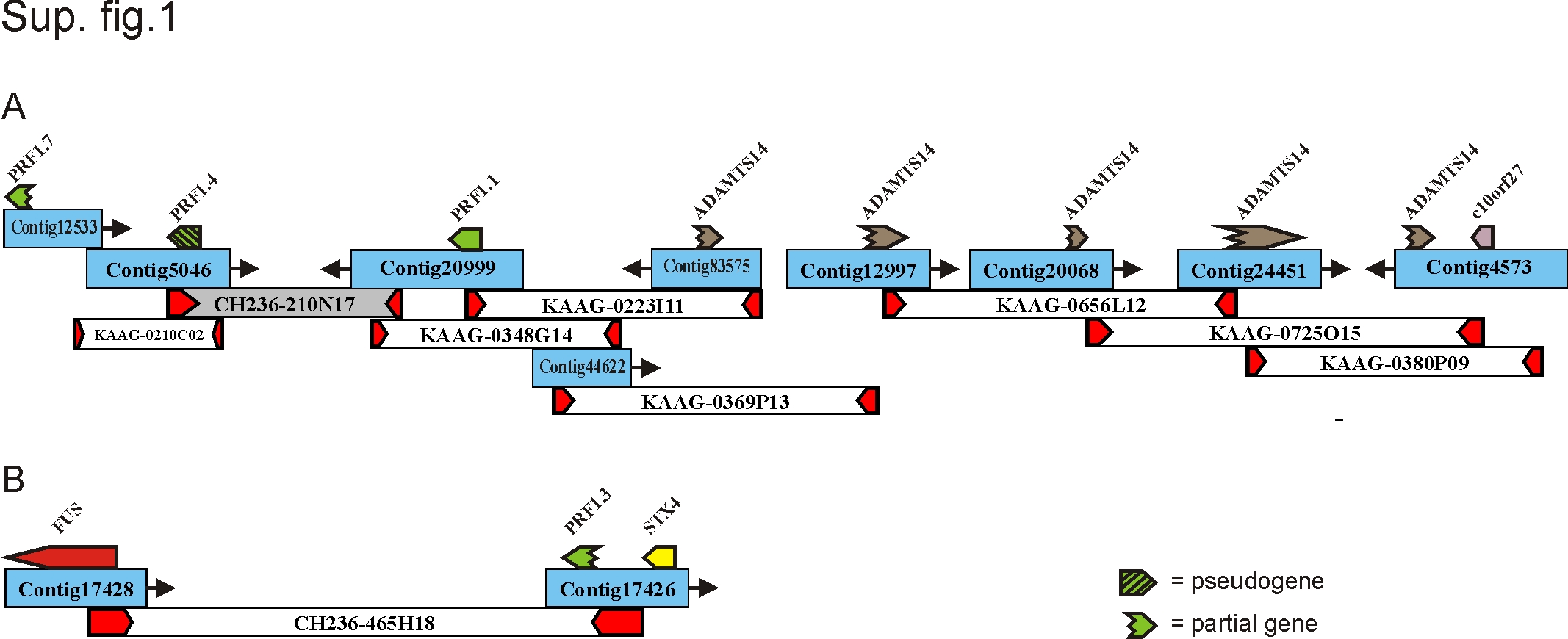

Supplement: Additional file 1 — Table S1. List of accessions used in this study. [file 1471-2148-12-59-S1.jpeg]
